# ﻿Lost for more than a century: the rediscovery of *Alsodesvittatus* (Philippi, 1902) (Anura, Alsodidae), one of the rarest and most elusive amphibians from Chile

**DOI:** 10.3897/zookeys.1230.135523

**Published:** 2025-03-06

**Authors:** Claudio Correa, Edvin Riveros-Riffo, Juan P. Donoso

**Affiliations:** 1 Laboratorio de Sistemática y Conservación de Herpetozoos (SyCoH), Departamento de Zoología, Facultad de Ciencias Naturales y Oceanográficas, Universidad de Concepción, Barrio Universitario S/N, Concepción, Chile Universidad de Concepción Concepción Chile; 2 Magíster(c) en Ciencias Biológicas mención Biodiversidad y Conservación, Instituto de Biología, Facultad de Ciencias, Universidad de Valparaíso, Valparaíso, Chile Universidad de Valparaíso Santiago Chile

**Keywords:** *Alsodesigneus*, *Alsodesneuquensis*, *Alsodesverrucosus*, Andean foothills, Chilean amphibians, conservation category, Rudolph Philippi, taxonomy

## Abstract

The legacy of the 19^th^-century naturalist Rudolph Philippi to the taxonomy of Chilean amphibians has been controversial since most of the species he described in 1902 have been questioned or invalidated. Here, we describe the rediscovery of *Alsodesvittatus*, a species that was not observed for 130 years after being collected, in three places very close to the type locality in the Andean foothills of the La Araucanía Region, Chile. The species was identified phenotypically by the vertebral line of some individuals, which turned out to be an intrapopulationally polymorphic trait. A phylogenetic analysis with mitochondrial genes, including most of the species of the genus, showed that the discovered populations of *A.vittatus* are paraphyletic with respect to the only individual of *A.neuquensis* included. We also describe populations from another area where *A.vittatus* was searched in the past, which we assigned here to *A.igneus* due to its geographic location and phylogenetic and phenotypic affinity. All these populations are part of two well-supported clades, but their relationships with nearby species (e.g., *A.norae* and *A.barrioi*) remain uncertain. These results ratify that the diversity and phylogenetic relationships of the genus in the Chilean Andes, particularly in the La Araucanía Region, are not yet well established. We discuss the possibility that *A.vittatus* and *A.neuquensis*, known until now only in Argentina, are the same species, and suggest downgrading the conservation status of *A.vittatus* from Critically Endangered to Endangered, considering the information from the new localities discovered.

## ﻿Introduction

Rudolph Amandus Philippi (1808–1904), of German origin, was one of the most important naturalists of Chile during the 19^th^ century (Kabat and Coan 2017). Philippi contributed enormously to the development of natural sciences in Chile in areas as diverse as zoology, botany, paleontology and geology. This work resulted in more than 450 publications (Taylor and Muñoz-Schick 1994), made during the more than five decades that he remained active. One of the most important positions he held was as director of the Museo Nacional de Historia Natural (MNHN) in Santiago, where he contributed to organizing and increasing the collections.

One of Philippi’s most enduring legacies is the extensive number of species of plants, animals, and fungi that he described, including more than 3300 species of plants (Muñoz-Schick et al. 2012), 2500 recent and fossil mollusks (Coan and Kabat 2017), and 800 species of insects (Camousseight 2005). It has been argued that the vast number of species that Philippi recognized is due to his adherence to the typological species concept prevailing at that time, differentiating many taxa by minimal phenotypic differences (Cei 1958; Castro et al. 2006). This is one of the main reasons Philippi’s contributions to the current taxonomy of many groups have been critically reviewed. Despite the criticism and progress in taxonomy over the last century, his contribution to the knowledge of Chile’s biota is undeniable. A century after his death, 1670 of the species he described were still considered valid, most of them plants (Castro et al. 2006). Among the animals, amphibians, phasmids (order Phasmatodea) and earwigs (Dermaptera) stand out, whose diversity as of 2006 included more than 10% of the species originally described by Philippi.

The revisions of the herpetozoans of Chile, which he carried out at a very late age, exemplify his taxonomic approach, by recognizing an excessively high number of species in comparison to previous studies. Thus, Philippi indicated that there were 45 species of snakes (Philippi 1899) and ~80 species of amphibians in Chile (Philippi 1902, *Suplemento a los batraquios chilenos descritos en la Historia Física i Política de Chile de don Claudio Gay*, in short, the “Suplemento”). Both works were critically reviewed: Donoso-Barros and Cárdenas (1965) focused on snakes, while Cei (1958) focused on amphibians. In the case of snakes, Philippi’s proposal proved to be totally unfounded, but in the case of amphibians, most species were considered synonymous or *incertae sedis* and only one (*Telmatobiuslaevis*) was considered valid by Cei (1958).

Subsequent studies revalidated some of the Philippi’s amphibian species, thanks to the collection of material from type localities (e.g., *Heminectesrufus* Philippi, 1902, currently *Rhinodermarufum*; Formas et al. 1975) or by the discovery of new populations that fit their descriptions (e.g., *Bufovenustus* Philippi, 1899, currently *Telmatobufovenustus*; Formas and Veloso 1982). Thus, a century after its publication, six species of amphibians described or redescribed by Philippi (1902) were recognized as valid (14.6% of the 41 species recognized at that time in Chile; Castro et al. 2006), although the magnitude of his contribution should be reevaluated, considering the taxonomic changes of the last two decades.

Among those six species are two of the genus *Alsodes*, whose taxonomic status is more controversial. *Alsodesverrucosus* (Philippi, 1902), originally described as *Borborocoetusverrucosus*, has a very vague type locality (the Andes of Cautín Province). It was first rediscovered in Argentina (Vellard 1947), and later, the population of Puyehue National Park, Chile (200 km south of the type locality) was assigned to this species without any explanation (Formas and Vera 1983). The type material of this species is probably lost (Formas 1995). The second species, *A.vittatus* (Philippi, 1902), has a more complex history. Roberto Donoso-Barros recovered from the MNHN some of the types used by Philippi to make his descriptions, including one of the three syntypes of *Cystignathusvittatus* (its original designation). However, Donoso-Barros (1976) considered that this specimen corresponded to *Eupsophusvertebralis* Grandison, 1961, so he proposed the new combination *Eupsophusvittatus*. This name appeared in several publications as referring to specimens of *Eupsophus* until Formas (1989a) demonstrated that the syntype recovered by Donoso-Barros corresponded to an *Alsodes*.

Unlike *A.verrucosus*, Philippi indicated a more precise type locality for *C.vittatus*, Hacienda San Ignacio de Pemehue (but see Material and methods), the collector (entomologist Philibert Germain) and the year in which he received the specimens (1894). Furthermore, the original drawing of the species was later found and published by Cei (1958). Despite all this information, there have been no sightings of the species after its description, and all more recent attempts (1995–2002) to find it have failed (Formas 1989a; IUCN SSC Amphibian Specialist Group 2016).

**Figure 1.**
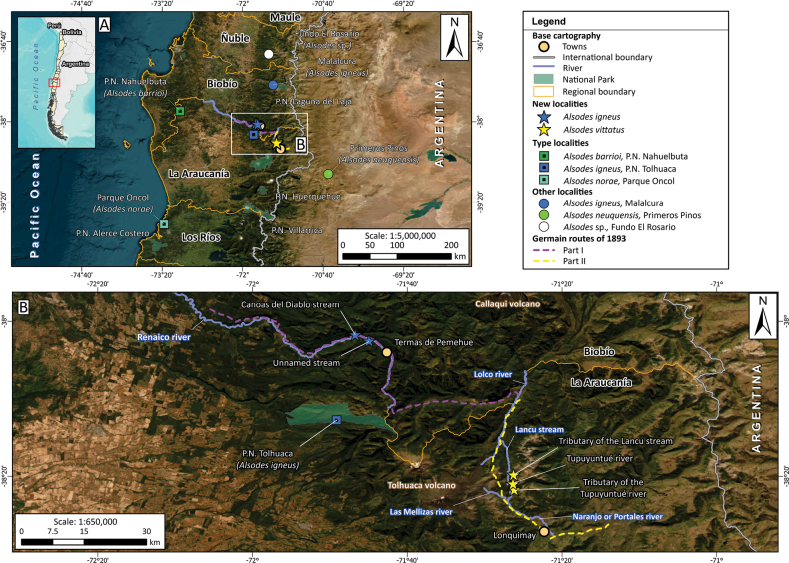
Location of the newly discovered populations of *Alsodesigneus* and *A.vittatus*, geographically and phylogenetically close species, and the reconstructed routes that Philibert Germain followed within the Hacienda San Ignacio de Pemehue in 1893 **A** populations of *Alsodes* between 36°40' and 40°S included in the phylogenetic analysis, including the new populations of *A.igneus* and *A.vittatus* described in this study **B** detail of map **A** showing the location of the newly discovered populations of *A.igneus* and *A.vittatus* (stars) and the routes (dashed lines) followed by Philibert Germain through the Hacienda San Ignacio de Pemehue according to Correa et al. (2023).

Recently, Correa et al. (2023) reconstructed the route that Philibert Germain possibly followed inside the Hacienda San Ignacio de Pemehue in late 1893, where he collected specimens of *Telmatobufovenustus* (Fig. 1B). The description of *A.vittatus* (Philippi 1902) specifies the same locality, collector and date as *T.venustus*, so we assumed that both species were collected at some point along that route, which served to guide our explorations.

In this study, we report the discovery of five localities of *Alsodes* located along or around the reconstructed route of Germain, three of which we assign to *A.vittatus* based on external morphological characteristics of adults and juveniles. We briefly describe these populations and investigate their phylogenetic relationships in the context of all the geographically closest species of the genus. Furthermore, we discuss the possible implications of this phylogenetic hypothesis for the taxonomy of *A.vittatus* and reevaluate the conservation category of this species, considering the information from the new localities discovered.

## ﻿Material and methods

### ﻿Explored areas

The type locality of *A.vittatus* (and *T.venustus*), Hacienda San Ignacio de Pemehue, was a huge *hacienda* (estate) located in the Andean zone of what is currently the southern end of the Biobío Region and the northern end of La Araucanía Region in Chile (~38°–38°30'S) (see the map in Klubock 2022). Philibert Germain described his journey through the estate in December 1893 (Germain 1894), which was reconstructed by Correa et al. (2023) (Fig. 1B). Germain (1894) indicated that he began his journey at the northwest end of the *hacienda*, where the landowner’s house was located, and then continued east along the north bank of the Renaico River. Currently, the public road runs along the southern bank of the Renaico River, whose valley initially runs approximately from west to east and, after passing the town of Termas de Pemehue, turns south. In 2015 and 2016 we explored some tributaries of the Renaico River along the road to Termas de Pemehue. In 2023 and 2024, we explored part of the final section of the reconstructed route of Germain’s journey (yellow dashed line on map in Fig. 1B). We departed from Lonquimay town towards the northwest, entering the valley of the Naranjo or Portales River, which has two tributaries, Las Mellizas and Tupuyuntué rivers. Finally, we followed the course of the Tupuyuntué River to the north, until we reached the valley of the Lancu stream (tributary of the Lolco River) in the adjacent sub-basin, located to the north.

### ﻿Samples and DNA extraction

We collected and/or sampled different numbers of specimens at different stages of development from five new localities (see details in Results). DNA was extracted from different types of tissue depending on the stage of development and whether the specimen was collected: thigh muscle or tongue for adults and juveniles, tail muscle for tadpoles, and buccal mucosa for one uncollected juvenile individual (unnamed stream). The buccal mucosa was obtained with a Copan 516CS01 swab and immediately dried with silica gel; the individual was released at the same capture site. The DNA was extracted with a commercial kit (Promega ReliaPrep™ gDNA Tissue Miniprep System) following the manufacturer’s instructions.

### ﻿PCR protocols and phylogenetic analysis

We obtained two mitochondrial fragments—one that extends between the 12S and 16S ribosomal genes (12S-16S), including the intervening tRNA-Val, and part of the cytochrome *b* (cytb)—to examine the phylogenetic affinities of the new populations. PCR protocols and primers to obtain these fragments are found in Correa et al. (2006), Correa et al. (2008) and Correa et al. (2013). We analyzed these two fragments together (concatenated) by Bayesian inference (BI), including one or a few specimens of most species of the genus (extracted from Faivovich et al. 2005; Blotto et al. 2013; Charrier et al. 2015; Correa et al. 2020). The BI analysis was performed with the program MrBayes v. 3.2.7a (Ronquist et al. 2012), applying independently to the fragment 12S-16S and to each codon position of cytochrome *b* the model-jumping option to explore the space of all General Time Reversible sub-models, plus gamma and proportion of invariable sites parameters. The analysis consisted of four independent chains run for 20 million generations, sampled every 1000 generations, conservatively discarding the first 25% of generations as burn-in after observing the stationarity of ln-likelihoods of trees in Tracer v. 1.7.1 (Rambaut et al. 2018). Convergence and mixing of chains were assessed by examining the average standard deviation of split frequencies (ASDSF), and expected sampling sizes (ESS) and the Potential Scale Reduction Factor (PSRF) for all parameters. The consensus tree was rooted with one specimen of *Eupsophuscalcaratus* (Günther, 1881), a representative of the sister genus to *Alsodes* (Blotto et al. 2013).

### ﻿Morphological descriptions

We described some external morphological characteristics of the collected adults and juveniles which were compared with those described for the geographically closest species from the Andean foothills—*A.igneus* Cuevas & Formas, 2005, *A.verrucosus* (Philippi, 1902), and *A.vittatus* (Philippi, 1902; Formas 1989a). Special emphasis was given to characters used in the diagnosis of these species, such as coloration patterns, shape of snout, interdigital membranes and other characteristics of the foot, and to their variation.

## ﻿Results

### ﻿New localities and sampled specimens

**Figure 2.**
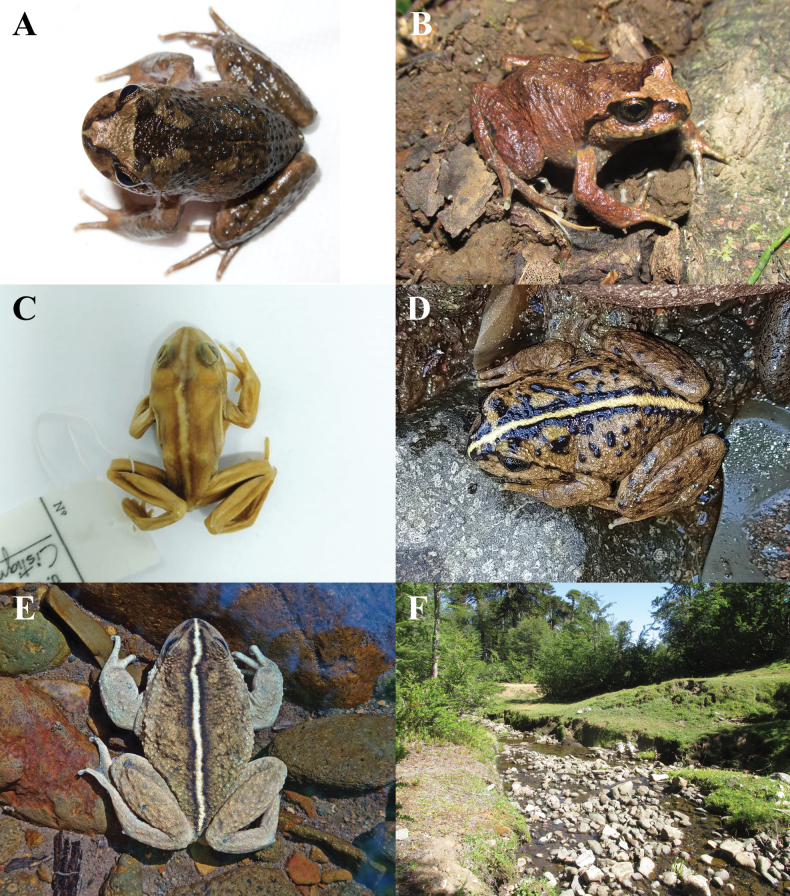
Individuals of *Alsodesigneus* and *A.vittatus* from the new populations, syntype of *A.vittatus* and environment of one of the new localities. The individual code used in the phylogenetic analysis is indicated in parentheses in Table 1**A** subadult male from Canoas del Diablo stream (Pem1j) **B** juvenile from the unnamed stream (not collected) (AS1j) **C** syntype of *A.vittatus*, Zoological Collection of the Museo de Concepción (MZUC), labeled as the holotype of “*Cistignathus*” *vittatus*, without collection number **D** juvenile from the tributary of the Lancu stream (AEL1j) **E** adult male from Tupuyuntué River underwater (RTp1m) **F** Tupuyuntué River at the point where tadpoles and juveniles of *A.vittatus* were observed.

We discovered five new localities of *Alsodes* along or near the route followed by Philibert Germain through the former Hacienda San Ignacio de Pemehue (Fig. 1B, Table 1). Two of these localities correspond to small water courses that flow north (Canoas del Diablo) and south of the Renaico River (unnamed stream). The first point is very close to a locality of *Eupsophusnodosus* (Duméril & Bibron, 1841) (*Alsodesnodosus*?) by Webb and Greer (1969). Both streams are located in an area dominated by temperate deciduous *Nothofagus* forest, although along the banks of the Renaico River the original vegetation has been partially eliminated or modified by human activities, or it has been replaced by forest plantations. The Canoas del Diablo stream has a steep slope, with a bed mainly made up of large rocks, and is surrounded by native forest. During two-night visits in spring 2015, several juveniles and numerous tadpoles were observed, and three juveniles were collected (Fig. 2A). The unnamed stream has a lower slope, with few rocks in its bed, and is located in an intervened area where native forest persists mainly along its bed. There, during the last of three-night visits made in the summer of 2016, only one small juvenile was observed on the leaf litter at the edge of the stream (Fig. 2B). The other three localities are situated at a higher altitude, in a temperate forest area where deciduous and resinous trees (*Araucariaaraucana*) are mixed. The tributary of the Lancu stream has a gentle slope, a mainly rocky bed, and is situated in a sparse forest with signs of human intervention. There, we explored at night ~250 m along the stream, and only found one adult female and one juvenile; we did not observe any tadpole. In contrast, in the Tupuyuntué River, we observed several adults, numerous juveniles and innumerable tadpoles at night, but only a few specimens were collected (Table 1). The river in the explored sector is rather a stream with a very slight slope, surrounded by a clearly intervened forest (Fig. 2F). Finally, we explored, during the day, a small section of a tributary of the Tupuyuntué River in an area almost completely cleared by human occupation, where we observed numerous tadpoles. In none of these last localities did we observe other species of amphibians. We observed a few adult individuals of *Eupsophus* sp. only in the two tributary streams of the Renaico River.

**Table 1.** New localities described in this study and specimens collected in each one. Altitudes according to Google Earth Pro. The specimen codes that appear in the Bayesian phylogenetic tree (Fig. 4) and in the text are indicated in parentheses.

Species	Locality	Latitude, Longitude	Altitude (m a.s.l.)	Collected/sampled specimens
*Alsodesigneus*	Canoas del Diablo stream	38°01'45"S, 71°46'44"W	631	Three juveniles (Pem1j-Pem3j)
*Alsodesigneus*	Unnamed stream	38°02'27"S, 71°44'57"W	674	One juvenile (not collected) (AS1j)
*Alsodesvittatus*	Tributary of the Lancu stream	38°19'49"S, 71°26'15"W	1491	One juvenile (AEL2j) and one adult female (AEL2h)
*Alsodesvittatus*	Tupuyuntué River	38°20'59"S, 71°26'19"W	1610	Two adult males (RTp1m and RTp2m), two juveniles and two tadpoles
*Alsodesvittatus*	Tributary of the Tupuyuntué River	38°21'44"S, 71°26'11"W	1421	Three tadpoles (ART1l)

### ﻿Description of the new populations of *Alsodesigneus*

According to the phylogenetic analysis, geographic location (Fig. 1A), and some morphological similarities, the populations from the tributaries of the Renaico River were assigned to *A.igneus*. Here, we describe the external morphology of the two largest specimens of the three collected in the Canoas del Diablo stream, and the coloration pattern of the small juvenile from the unnamed stream. The largest specimen (SVL = 42.5 mm) from Canoas del Diablo can be considered a subadult male because it has patches of spines on the chest and poorly developed spines on fingers 1 and 2 of the hand (not cornified) (Fig. 2A). It has the canthus rostralis and postocular fold (supratympanic fold) well-developed, short and rounded snout in dorsal and lateral view, smooth skin with small granulations on the dorsal surface of body and limbs, ventral surface of thighs with numerous small tubercles, toes with reduced lateral fringes and poorly developed interdigital membranes. Another smaller juvenile (SVL = 34.5 mm) is morphologically similar to the previous one but has a less developed canthus rostralis and postocular fold, and lacks spines on the chest and fingers. Both specimens in life had a light brown background color with a continuous dark brown spot on the anterior part of the back. This spot included a triangle that converges towards the back, with its base between the eyes, which is flanked on both sides by elongated, lighter scapular spots (Fig. 2A, B). Additionally, they had other small, irregular dark spots scattered over the back and limbs and a lighter triangle on the head. This lighter triangle was delimited by the base of the dark dorsal triangle and two dark bands extending from the tip of the snout to the eyes, beneath the canthus rostralis. The belly was whitish with small, diffuse brown spots, and the iris was black with golden reticulations. The smaller juvenile (SVL = 32 mm) from the unnamed stream is similar to the previous two but has a reddish-brown color (Fig. 2B). The lateral band on the head extends beyond the eye to near the base of the arm, and its belly is leaden, speckled with small, pale yellowish spots. Therefore, similarities in coloration patterns, as well as in the development of the lateral fringes of the toes and interdigital membranes, reinforce the idea that the new populations can be assigned to *A.igneus* (Cuevas and Formas 2005).

### ﻿Description of the new populations of *Alsodesvittatus*

**Figure 3.**
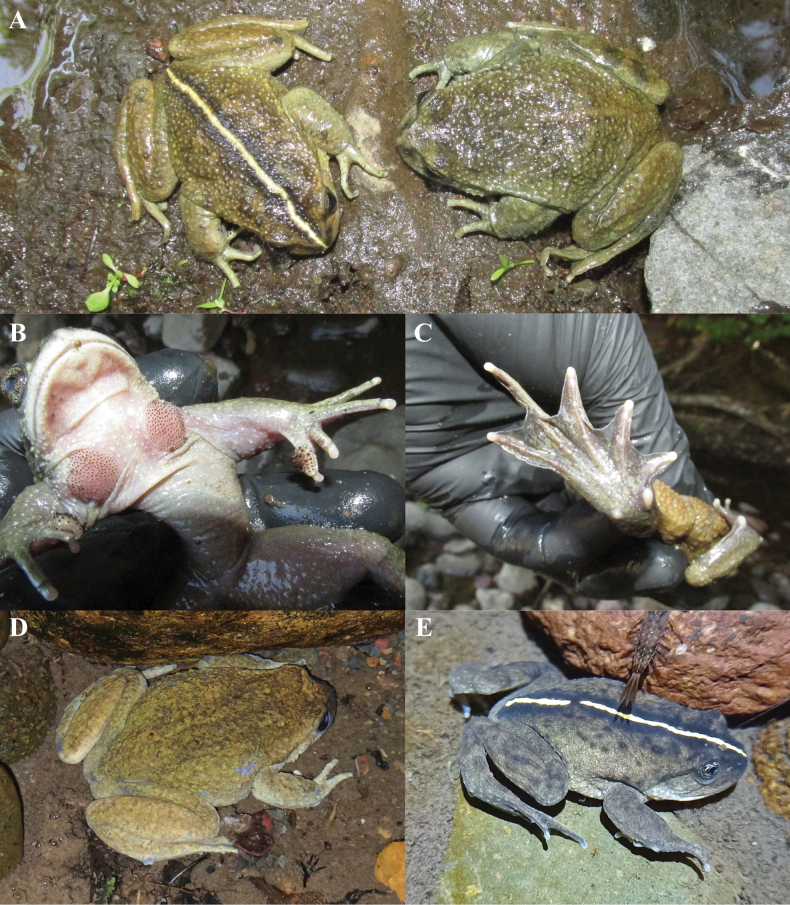
Some morphological characteristics of *Alsodesvittatus***A** two adult males from the Tupuyuntué River showing the polymorphisms of vertebral line and coloration patterns (left, RTp1m; right, RTp2m) **B** secondary sexual characters (thickened forearms, patches of spines on chest and spines on fingers 1 and 2) of the male RTp2m **C** plantar view of the right foot of the same male **D** adult female from the Tupuyuntué River underwater (not collected) **E** juvenile from the Tupuyuntué River underwater (SVL = ~40 mm).

The new populations of *A.vittatus* were assigned to this species due to their proximity to the route followed by the original collector (Germain) through the type locality (Fig. 1B), as well as the presence of the species’ most conspicuous characteristic, the vertebral line (Figs 2, 3). The external morphology, color patterns and variation of adult and juvenile specimens from two of three of the discovered localities are described here and compared with the original descriptions of *A.vittatus* and *A.verrucosus* (Philippi 1902). The two specimens from the tributary of the Lancu stream—a juvenile (AEL1j, Fig. 2D) and an adult female (AEL2h)—show that *A.vittatus* exhibits variation in coloration patterns and a polymorphism in the presence/absence of the vertebral line. The juvenile has a well-defined yellowish vertebral band bordered by black. The dorsal background color is brown, with numerous granulations on the back and limbs, most of which are pigmented black. The ventral surface is pinkish-brown (greyish on the abdomen), with diffuse yellowish reticulations. The ventral surface of the thighs has numerous small, densely arranged granulations. The snout is slightly pointed in dorsal view and slightly truncated in lateral view. The toes are fringed, and the interdigital membranes are poorly developed. The color pattern of the female differs markedly: the vertebral line is absent, the dorsal background color is light brown, and the skin is smooth with irregular dark brown spots on the back. The largest dorsal spot anteriorly has an inverted triangular shape, with one of its vertices pointing backwards and the opposite side located between the eyes. There are numerous small granulations scattered over the dorsal surface. The ventral color is whitish with a slight pinkish tint, particularly under the thighs, where the ventral surface has numerous, dense, small granulations. The snout is short, rounded in dorsal view, and truncated in lateral view. The canthus rostralis is poorly developed, while the postocular fold is thick and extends to the base of the arm. The toes have well-developed lateral fringes and more extensive interdigital membranes than those of the juvenile.

**Figure 4.**
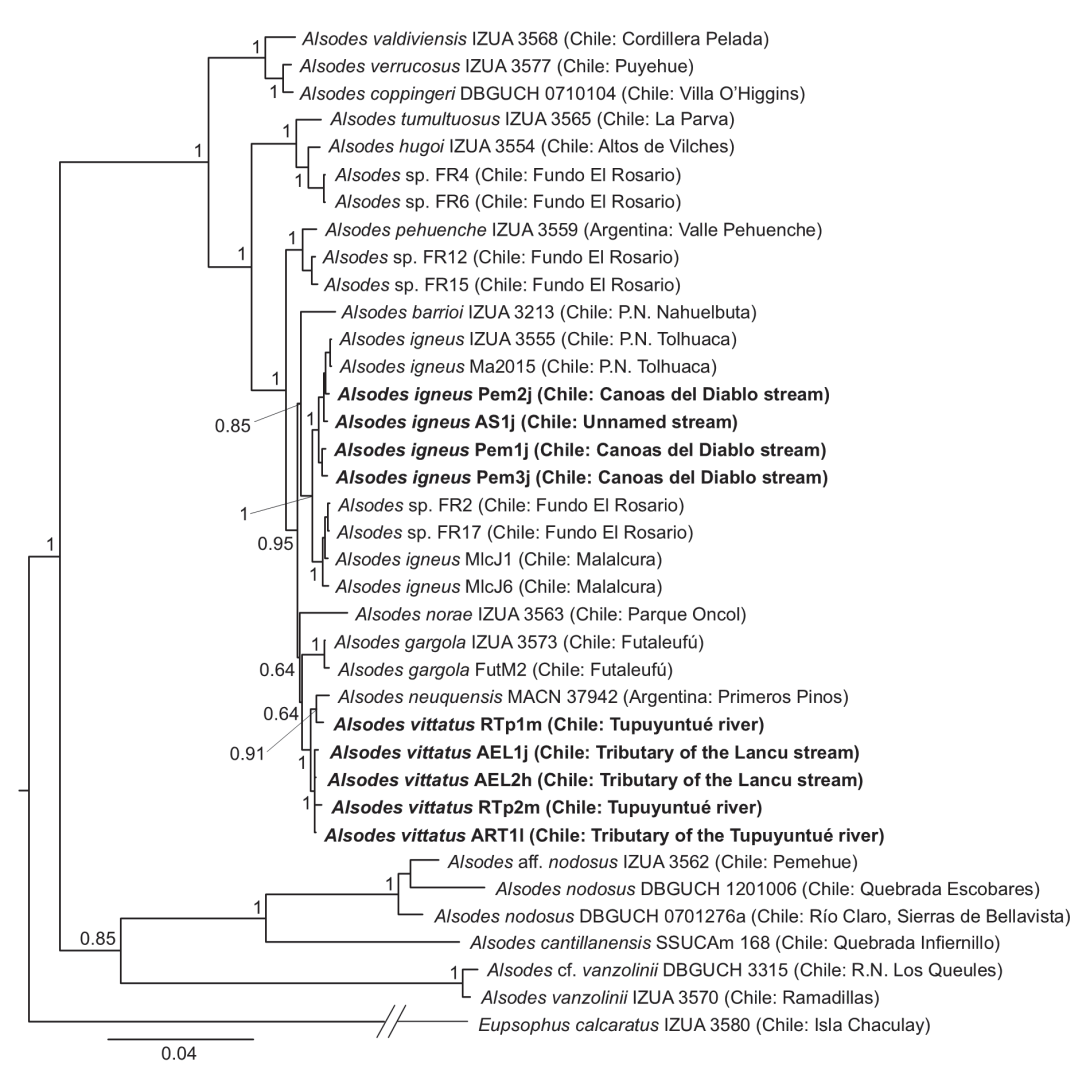
Bayesian consensus tree (50% majority-rule) of the two concatenated mitochondrial fragments, showing the relationships of the new populations within the genus *Alsodes*. Individuals from the new populations are highlighted in bold (see details of the codes in Table 1). Numbers next to the nodes correspond to posterior probabilities and the scale bar below the tree represents the expected substitutions per site along the branches.

The two adult males from the Tupuyuntué River (RTp1m and RTp2m; Fig. 3A) exhibit the secondary sexual characteristics that define the genus (Fig. 3B) and confirm the polymorphism of the vertebral line within the species. They also extend the range of variation in the species’ coloration patterns: male RTp1m is light brown with light yellow and green tints, while male RTp2m is olive brown. We also observed a female of similar size and coloration to male RTp2m (not collected; Fig. 3D). In both males, the dorsal coloration is darker with very diffuse dark spots; however, male RTp1m has a darker color around the vertebral line. Both males have numerous granulations on the dorsal surface and skin folds on the sides, which are more developed in male RTp2m. They both possess a rounded snout in dorsal and lateral view, a slightly accentuated canthus rostralis, and a well-developed postocular fold. They have toes with lateral fringes and well-developed interdigital membranes, although these are emarginate (Fig. 3C). Both individuals have black irises with golden reticulations.

Apart from the vertebral line, there are few external features in the original description of *A.vittatus* that can be compared with those of the newly collected specimens. In fact, there is a significant difference in the coloration pattern. According to Philippi (1902), “the upper part of the body” of *A.vittatus* “is more or less intense black”. Most of the individuals that we observed do not fit this description, as the dorsal coloration of the juvenile AEL1j and adult individuals ranges from olive brown to pale brown, with either indistinct or well-marked dark spots. In the existing syntype (Fig. 2C), the only dark area preserved is that bordering the vertebral line. One possibility is that the specimens described by Philippi were already darkened due to fixation in alcohol, as he mentions this detail (the specimens we collected darkened when preserved in ethanol). However, we observed several juveniles (~35–40 mm) in the Tupuyuntué River exhibiting dark brown dorsal coloration and a vertebral line, which align with the size reported by Philippi for the species (“trunk length 41 mm”; Fig. 3E). Therefore, Philippi’s description may have been based on subadult individuals with such coloration. The greatest coincidence is observed in the coloration of the palms and soles, which Philippi described as blackish. All the individuals we captured exhibit darker ventral surfaces on the feet and hands (Fig. 3C). Additionally, the geographic location of the new populations coincides with the area where *A.verrucosus* was described; however, there are few external morphological characteristics for comparison in this case. According to Philippi (1902), this species lacks the vertebral line but is blackish above and reddish-brown below. While there is no coincidence in color, some individuals observed in the Tupuyuntué River (e.g., male RTp2m) have “the back densely covered with soft warts” (although small), as described by Philippi for this species. Therefore, it cannot be ruled out that Philippi’s description pertains to specimens similar to those observed in the Tupuyuntué River, although darker. However, the resolution of the taxonomic status of *A.verrucosus* is beyond the scope of this study.

### ﻿Phylogenetic relationships and taxonomic issues

The sequences obtained in this study were deposited in GenBank (fragment 12S-16S: PQ800373–PQ800387; cytb: PQ800388–PQ800401). We obtained final alignments of 2001 nucleotide sites for the fragment 12S-16S and of 953 for the cytb. The cytb alignment was almost complete due to a few slightly shorter sequences and because the only available *A.neuquensis* sequence is only 385 base pairs long (Faivovich et al. 2005; only 361 sites from this species were aligned). Our phylogenetic reconstruction is congruent with previous hypotheses, identifying two main clades within the genus with high support. One clade comprises *A.cantillanensis* Charrier, Correa, Castro & Méndez, 2015, *A.nodosus*, and *A.vanzolinii* (Donoso-Barros, 1974), while the other includes all remaining species (e.g., Blotto et al. 2013; Charrier et al. 2015; Correa et al. 2020). Within the latter clade, several groupings have been consistently recovered in these studies. One such grouping includes species known from the Coastal Mountains of Chile and the Andean foothills of Chile and Argentina between 37°15' and 39°45'S (*A.barrioi* Veloso, Díaz, Iturra & Penna, 1981, *A.igneus*, *A.neuquensis*, and *A.norae* Cuevas, 2008), as well as *A.gargola* Gallardo, 1970, which extends further south between 40°25' and 44°25'S, mainly in Argentina and marginally in Chile (Mella-Romero et al. 2022). All populations described here are included within this clade, but they are related to different nominal species. The two populations located on either side of the Renaico River are closely related to the known populations of *A.igneus* and are geographically situated between them. Furthermore, the specimens are phenotypically similar to *A.igneus* (see above), leading us to assign them to that species. On the other hand, populations identified as *A.vittatus* based on morphology and geographical location are closely related to *A.neuquensis*. In fact, a specimen from the Tupuyuntué River (the male with the vertebral line, Figs 2E, 3A) is more related to *A.neuquensis* than to another male specimen from the same population. In this case, we did not make a taxonomic decision due to the lack of additional material for *A.neuquensis* for morphological comparisons and a more complete molecular phylogenetic analysis. However, we emphasize the great morphological similarity between the male from Tupuyuntué related to *A.neuquensis* and a specimen from the vicinity of Primeros Pinos (east of the type locality of *A.neuquensis*), which also exhibits a vertebral line that was identified by Cei (1987) and Lavilla and Cei (2001) as *A.verrucosus* (see color photo in this last source). Consequently, Cei (1987) assumed that *A.neuquensis* (as *A.gargolaneuquensis*) and *A.verrucosus* coexist in Primeros Pinos. More recent observations allow us to interpret the coexistence of these two species in that place as a polymorphism of presence/absence of the vertebral line in *A.neuquensis* (Carmen Úbeda, personal communication). Therefore, the absence of reciprocal monophyly (at least of mitochondrial DNA) and morphological affinity (coloration patterns and polymorphism of the vertebral line) between *A.vittatus* and *A.neuquensis* indicate that it is necessary to evaluate the possible conspecificity of these two species. Finally, it is necessary to emphasize that the relationships among the lineages made up of *A.barrioi*, *A.norae*, *A.gargola*, *A.igneus* and *A.vittatus* + *A.neuquensis* are not well resolved (low support values) as observed in previous studies (e.g., Blotto et al. 2013; Charrier et al. 2015).

## ﻿Discussion

More than 120 years after its publication, Philippi’s “Suplemento” continues to have a significant influence on the current taxonomy of Chilean amphibians. Editorial issues (such as the non-publication of the figures), museological problems (the loss of most of the types), and taxonomic challenges (e.g., numerous species that are not currently recognizable) (Formas 1995) led to the near-complete dismissal of this work (Cei 1958). However, later discoveries confirmed the validity of some of the described species. The rediscovery of *A.vittatus*, after 130 years since the collection of the type specimens, demonstrates that Philippi’s taxonomic legacy has not yet been fully dimensioned.

The data and material available for *A.vittatus* are exceptional among the amphibian species described by Philippi. Firstly, it is one of the few species in the “Suplemento” that has a precisely defined type locality, as well as the name of the collector and year of collection. Although, in this case, the type locality turned out to be extraordinarily broad, a detailed investigation of this information and other historical documents enabled the identification of the route followed by Germain in 1893 (Fig. 1B; see details in Correa et al. 2023) and so a more specific area where the species was finally found. Secondly, the most conspicuous external characteristic originally described for *A.vittatus*—”a white or yellow band, which runs from the tip of the snout to the anus” (from which its name is derived; Lavilla 2021)—is clearly observed in Philippi’s original drawing, which was published later (Cei 1958). This characteristic facilitated its unambiguous identification in the field. Thirdly, *A.vittatus* is one of the few amphibian species described by Philippi (1902) for which a type specimen has survived, specifically one of the three original syntypes. The careful examination of this specimen (Fig. 2C) allowed Formas (1989a) to definitively clarify that the species belongs to the genus *Alsodes*. All this information together provides a stronger evidential basis to support the validity of *A.vittatus* compared to other taxa from the same area described by Philippi (1902) that would be congeneric (e.g., *Borborocoetusverrucosus* and *B.andinus*).

As previously mentioned, the most distinctive external feature of *A.vittatus* is its vertebral line, observable in the only remaining syntype, as well as in some recently collected specimens. However, our molecular data reveal that this line represents an intraspecific polymorphism, which we verified in two newly described populations, despite the low number of individuals observed. The polymorphism of presence/absence of the vertebral line has been documented in several species of *Alsodes* (e.g., *A.gargola*, Gallardo 1970; *A.hugoi* Cuevas & Formas, 2001; Correa et al. 2018; *A.verrucosus* from Puyehue, Chile, Charrier 2019, and from Pucará, Lake Lácar, Argentina; Cei 1980; *A.neuquensis*, Carmen Úbeda, personal communication), as well as in other genera inhabiting Chile, such as *Eupsophus* (notably within the species of the *vertebralis* group; Formas 1989b) and *Pleurodema* (e.g., *P.thaul* (Schneider, 1799), Cei and Capurro 1957). Therefore, despite the importance that Philippi gave to this feature in the description of the species, our observations allow us to rule it out as a diagnostic character.

Another distinctive feature of the description of *A.vittatus*, evident in the original drawing, is the more or less intense black color of the upper part of the body. We observed juvenile individuals with dark brown coloration and a vertical line very similar to that depicted in the drawing (Fig. 3E) and with a size similar to the SVL reported by Philippi (1902) for the species (41 mm). This suggests that the description may have been based on immature specimens with that coloration. In addition to these dark-colored juveniles, we observed adults (males and females) and juveniles with different color patterns coexisting in two locations, but despite the low number of individuals observed, we did not find an association between certain colorations and developmental stage and/or sex. We also did not observe whether individuals changed color or whether coloration was associated with certain microhabitat characteristics (for example, lighter or darker substrates), so we interpret the variation in coloration as intra- and interpopulation polymorphism. However, we cannot rule out the possibility of ontogenetic variation and that the different color patterns are transient responses to thermoregulatory requirements or strategies for camouflage in environments with different degrees of illumination, as has been described in other anurans (e.g., Kang et al. 2016, Park et al. 2023).

From a phylogenetic perspective, all the new populations described here are grouped with *A.barrioi*, *A.igneus*, *A.gargola*, *A.neuquensis* and *A.norae*, a clade recognized since Blotto et al. (2013), which makes biogeographical sense. Although the relationships among these species remain unsolved, the populations along the road to the Termas de Pemehue locality are more closely related to *A.igneus*, while the populations of *A.vittatus* are grouped with *A.neuquensis*, in both cases with high support. In the first one, the taxonomic decision seems uncontroversial, but the second poses a more complex situation. The specimens from the populations that we identify as *A.vittatus* are paraphyletic with respect to the only specimen of *A.neuquensis* included in the analysis. Although this may be due in part to using only mitochondrial evidence and a shorter fragment of cytochrome *b* from *A.neuquensis*, the high morphological similarity between the male with vertebral line from Tupuyuntué River (Figs 2E, 3A) and the specimen from the vicinity of Primeros Pinos (identified as *A.verrucosus* by Cei 1987; Lavilla and Cei 2001) is evident. Therefore, morphological similarity, shared vertebral line polymorphism, lack of reciprocal mitochondrial monophyly, and geographic proximity suggest that *A.vittatus* and *A.neuquensis* could be the same species.

The IUCN lists *A.vittatus* as Data Deficient, recognizing that it has not been observed again in more than 100 years and that there is no population, ecological, distributional, or threat information available for this species. However, the official classification system of the Government of Chile, made official by Supreme Decree No. 42/2011 of the Ministry of the Environment, considers it Critically Endangered due to its reduced distribution, restricted to the type locality, and the deterioration of its habitat due to agricultural and forestry activity. The latter is an observation by Formas (1989a) referring to the area of ​​the Termas de Pemehue, where according to our data, only *A.igneus* is found, so the current Chilean categorization refers to this taxon. Therefore, it is necessary to review whether the available information from the newly discovered populations of *A.vittatus* is sufficient to reevaluate its conservation category under the criteria of IUCN.

The maximum distance between the new three localities of *A.vittatus* is ~3.5 km in a straight line (Fig. 1), but they are located in the headwaters of two different hydrographic sub-basins. Two of these localities are directly hydrographically connected, so according to the IUCN guidelines, they could be considered only two localities. The three original localities are arranged in a line with a north-south direction, so it is difficult to estimate the extent of occurrence; however, due to their distances, the area of occupancy can be approximated to be 8 km^2^. Applying criterion B, this falls within the limits to classify it as Critically Endangered, but the condition of a severely fragmented range, or number of localities equal to one can be ruled out (condition a). There is also no indication that the range, population numbers, or abundance are fluctuating extremely (condition c), but our observations suggest that criterion b can be applied cautiously.

The new populations are found in montane forests dominated by *Nothofagus* and *Araucariaaraucana*. Satellite images show that the forest is continuous only in the highest parts of these mountain systems (at the sources of the valleys) and highly fragmented in the lower parts of the valleys (such as Naranjo or Portales River). We were able to confirm that there is timber exploitation and livestock activity throughout the entire valley, and the presence of introduced salmonids (trout) in the highest flow rivers throughout the area. Two other recognized threats in the area are forest fires and the presence of *Didymospheniageminata* (didymo) in some Andean water systems (reported in state reports). In addition, testimonies from local residents indicate that summers are becoming increasingly hotter in the area, with less snow accumulation in winter. This suggests that climate change could constitute an additional threat. Therefore, considering all these factors, we infer a continuing decline in habitat quality and recommend evaluating this species as Endangered based on the B2ab(iii) criterion.

In summary, the rediscovery of *A.vittatus* shows that there are still aspects of Philippi’s work that need to be revised. Furthermore, it confirms the limited biogeographic knowledge of amphibians in this area of the Andes, particularly concerning *Alsodes*, as the new populations were found in an extensive region where there were no previous records of the genus (compare with Correa et al. 2018). Findings like these demonstrate that a greater exploration effort, guided by rigorous historical research, is required to continue revealing the diversity and distribution patterns of Chile’s amphibians.

## ﻿Additional information

### Author ORCIDs

Claudio Correa ￼ https://orcid.org/0000-0002-2081-3565

### Data availability

All of the data that support the findings of this study are available in the main text.
